# Deferiprone–resveratrol hybrid attenuates iron accumulation, oxidative stress, and antioxidant defenses in iron-loaded human Huh7 hepatic cells

**DOI:** 10.3389/fmolb.2024.1364261

**Published:** 2024-03-20

**Authors:** Jin Li, Pimpisid Koonyosying, Woranontee Korsieporn, Narisara Paradee, Nuntouchaporn Hutachok, Honghong Xu, Yongmin Ma, Hataichanok Chuljerm, Somdet Srichairatanakool

**Affiliations:** ^1^ Department of Biochemistry, Faculty of Medicine, Chiang Mai University, Chiang Mai, Thailand; ^2^ Department of Biochemistry, Faculty of Basic Medicine, Youjiang Medical University for Nationalities, Baise, China; ^3^ School of Pharmaceutical and Chemical Engineering, Taizhou University, Taizhou, China; ^4^ School of Health Sciences Research, Research Institute for Health Sciences, Chiang Mai University, Chiang Mai, Thailand

**Keywords:** iron chelator, deferiprone, resveratrol, antioxidant, hepatoprotective

## Abstract

Chronic liver diseases are complications of thalassemia with iron overload. Iron chelators are required to remove excessive iron, and antioxidants are supplemented to diminish harmful reactive oxygen species (ROS), purposing to ameliorate oxidative liver damage and dysfunctions. The deferiprone–resveratrol hybrid (DFP–RVT) is a synthetic iron chelator possessing anti-β-amyloid peptide aggregation, anti-malarial activity, and hepatoprotection in plasmodium-infected mice. The study focuses on investigating the antioxidant, cytotoxicity, iron-chelating, anti-lipid peroxidation, and antioxidant defense properties of DFP–RVT in iron-loaded human hepatocellular carcinoma (Huh7) cells. In the findings, DFP–RVT dose dependently bound Fe(II) and Fe(III) and exerted stronger ABTS^•^- and DPPH^•^-scavenging (IC_50_ = 8.0 and 164 μM, respectively) and anti-RBC hemolytic activities (IC_50_ = 640 μM) than DFP but weaker than RVT (*p* < 0.01). DFP–RVT was neither toxic to Huh7 cells nor PBMCs. In addition, DFP–RVT diminished the level of redox-active iron (*p* < 0.01) and decreased the non-heme iron content (*p* < 0.01) in iron-loaded Huh7 cells effectively when compared without treatment in the order of DFP–RVT > RVT ∼ DFP treatments (50 µM each). Moreover, the compound decreased levels of hepatic ROS in a dose-dependent manner and the level of malondialdehyde, which was stronger than DFP but weaker than RVT. Furthermore, DFP–RVT restored the decrease in the GSH content and GPX and SOD activities (*p* < 0.01) in iron-loaded Huh7 cells in the dose-dependent manner, consistently in the order of RVT > DFP–RVT > DFP. Thus, the DFP–RVT hybrid possesses potent iron chelation, antioxidation, anti-lipid peroxidation, and antioxidant defense against oxidative liver damage under iron overload.

## 1 Introduction

Iron is critical in many cellular processes of all living organisms; however, excessive iron catalyzes the production of reactive oxygen species (ROS) through Haber–Weiss and Fenton reactions (de Lima et al., 2019). Consequently, ROS levels exceeding the cellular antioxidant defensive mechanisms can cause oxidative damage and dysfunctions of vital organs, including the heart, liver, pancreas, and endocrine glands (Goswami et al., 2002). Excess iron accumulation is the most common in the spleen and liver of β-thalassemia patients (Dev and Babitt, 2017). Accordingly, many thalassemia patients suffer from liver inflammation and eventually die of hepatocellular carcinoma (Llaurado et al., 2020) when they are coincidently infected with the hepatitis virus (Fernandez-Real et al., 2002). Since large amounts of iron are massively deposited in ferritin, hemosiderin, and redox-active labile iron pools (LIPs) in hepatic parenchymal cells, these patients have shown clinical signs and symptoms, including hepatomegaly, jaundice, liver inflammation and cirrhosis, and increased liver enzyme levels (Lee et al., 2010; Pinto et al., 2013).

Iron chelators must chelate redox-active iron in plasma and tissues and iron deposited in tissues, and resulting iron chelates will be excreted via the hepatobiliary duct into the small intestine and finally removed from the body via urine and feces (de Lima et al., 2019). Accordingly, a parenterally administered desferrioxamine (DFO), deferiprone (DFP), and deferasirox (DFX) are required to remove toxic iron and alleviate organ dysfunctions, including the liver (Das et al., 2015). Antioxidants function to remove ROS, inhibit ROS-induced lipid peroxidation, and reinforce antioxidant defense systems, such as reduced glutathione (GSH), glutathione peroxidase (GPX), thioredoxin reductase (TrxR), catalase (CAT), and superoxide dismutase (SOD) in cells. DFP was found to inhibit lipid peroxidation in plasmodium-infected red blood cells (RBCs) in the liver from chemical-fed rats (Sadrzadeh et al., 1994; Cerna et al., 2011; Chuljerm et al., 2021; Maneekesorn et al., 2021). Importantly, DFP can prevent lipid peroxidation and restore GPX activity in the oxidative liver from tamoxifen-induced rats (Cerna et al., 2011). Similarly, DFP attenuates lipid peroxidation and increases GSH but not the GPX activity in the liver of trimethylhexanoyl (TMH)–ferrocene-fed mice (Eybl et al., 2002). Surprisingly, not only DFP but also naringin, quercetin, and myricetin can restore the decreased GPX and TrxR activities in the liver of iron-loaded rats (Hodkova et al., 2010). Interestingly, resveratrol (RVT), which is a natural phytoalexin present in grape skin and red wine, has antioxidant, anti-lipid peroxidation, and hepatoprotective effects by improving the decreased levels of SOD, CAT, and GPX in the liver of ethanol-fed rats (Kasdallah-Grissa et al., 2007). In contrast, RVT treatment (10 mg/kg/day) did not influence hepatic SOD and CAT activities and oxidative stress in high-fructose diet-induced rats (Yilmaz Demirtas et al., 2018).

Hence, novel iron chelators are being developed to achieve more effectiveness but fewer adverse effects. Thereafter, the deferiprone–resveratrol hybrid (DFP–RVT), in which the chelating moiety DFP and natural antioxidative RVT were merged in one molecule, was previously synthesized and showed similar pFe(III) values to that of DFP (19.16 and 20.60, respectively) and more lipophilicity than DFP as well (Xu et al., 2017; Maneekesorn et al., 2021). Nevertheless, it is not clear whether DFP–RVT has side effects since we did not perform the toxicity study at that time. Importantly, DFP–RVT showed metal-chelating properties, greater ROS-scavenging activity than Trolox, a better inhibitory effect against Cu^2+^/Fe^3+^-induced β-amyloid-(1–42) protein aggregation than RVT, anti-malarial activity against the growth of *Plasmodium falciparum* culture and *Plasmodium berghei* in mice, and anti-lipid peroxidation and hepatoprotection in the *P. berghei*-infected mice (Xu et al., 2017; Chuljerm et al., 2021; Chuljerm et al., 2022; Kampoun et al., 2023). Hypothetically, DFP–RVT could increase antioxidative and iron-chelating properties for greater improvement of oxidative stress and inflammation in iron-loaded liver cells. Therefore, we aim to examine the toxicity and ROS-scavenging and iron-chelating activities of DFP–RVT in iron-loaded human hepatocellular carcinoma (Huh7) cells.

## 2 Materials and methods

### 2.1 Chemicals and reagents

Acetic acid, L-ascorbic acid (AA), 2,20-azino-bis(3-ethylbenzthiazoline-6-sulfonic acid (ABTS), bovine serum albumin (BSA), 3-(4,5-dimethylthiazol-2-yl)-2,5-diphenyltetrazolium bromide (MTT) reagent, catechin, deferiprone (DFP), 2′,7′-dichlorofluorescein diacetate reduced form (DCFH-DA), dimethyl sulfoxide (DMSO), 2,2-diphenyl-1-picrylhydrazyl (DPPH), ferrozine, DTNB = 5,5-dithiobis (2-nitrobenzoic acid), ferric chloride, fetal bovine serum (FBS), ferric ammonium citrate (FAC), ferrous ammonium sulfate (FAS), Folin–Ciocalteu reagent, 3-(N-morpholino) propane sulfonic acid (MOPS), metaphosphoric acid (MPA), nitrilotriacetic acid (NTA), penicillin G, potassium persulfate, streptomycin, thiobarbituric acid (TBA), trichloroacetic acid (TCA), 1,1,3,3-tetramethoxypropane (TMP), 6-hydroxy-2,5,7,8-tetramethylchroman-2-carboxylic acid (Trolox), 1(2-(4-iodophenyl)-3-(4-nitrophenyl)-5-(2,4-dithiophenyl)-2H-tetrazole, and monosodium salt (WST) were purchased from Sigma-Aldrich Chemicals Company (St. Louis, MO, United States of America). 2,2′‐Azo‐bis(2‐amidinopropane) dihydrochloride (AAPH) was acquired from Merck KGaA Group, Darmstadt, Germany. DFO, desferrioxamine mesylate (Desferal^®^) was obtained from a local drug store at Maharaj Nakorn Chiang Mai Hospital, Faculty of Medicine, Chiang Mai University, Thailand. Fetal calf serum (FCS), phosphate-buffered saline (PBS), Dulbecco’s modified Eagle's medium (DMEM), and tryptic soy agar (TSA) were purchased from Invitrogen (GIBCO™), a division of Thermo Fisher Scientific Corporation located in Waltham, MA, United States of America. A superoxide dismutase (SOD) assay kit was procured from Sigma-Aldrich Chemical Company (St. Louis, MO, United States of America). Glutathione concentration (Catalog number E-BC-K096-S) and glutathione peroxidase (Catalog number E-BC-K030-S) activity assay kits were purchased from Wuhan Elabscience Biotechnology Company Limited, Hubei, People’s Republic of China. Human hepatocellular carcinoma (Huh7) cells were provided by Thermo Fisher Scientific Company in Waltham, MA, United States of America. HPLC- or Analar-grade organic solvents, including ethyl acetate, absolute ethanol, and methanol, were procured from BDH Chemicals Company, Poole, UK. DFP–RVT was synthesized by Ma et al., and the synthetic method was described previously (Xu et al., 2017).

### 2.2 Human ethics

Blood collection protocol was submitted for permission by the Director of Maharaj Nakorn Chiang Mai Hospital and expeditiously reviewed by the Ethical Committee for Human Study of the Faculty of Medicine, Chiang Mai University, Chiang Mai, Thailand. Informed consent was also provided by recruited healthy volunteers. Human ethics approval (Study Code: BIO-2565-09172/Research ID: BIO-2565-09172, 27 October 2022) was obtained before the commencement of blood collection.

### 2.3 Assay of iron-binding activity

This study uses the method of Pangjit et al., and the method description partly reproduces their wording (Pangjit et al., 2015). In principle, iron chelator specifically binds ferrous (Fe^2+^) and ferric (Fe^3+^) ions using coordination bonds and forms a colored iron chelate. Thus, DFP and DFP–RVT (100 μM) are incubated with FAC or FAS (12.5, 25, 50, and 100 μM each) for 30 min, and absorbance (A) values at the wavelength range of 200–800 nm are measured against a reagent blank using a double-beam UV-VIS spectrophotometer. The wavelength illustrating maximal absorption (λ_max_) of the colored product was recorded and used in other experiments. In a dose–response study, the FAC (62.5 μM each) solution was incubated with different concentrations of DFP and DFP–RVT for 1 h, and A-values were photometrically measured at the λ_max_ value.

### 2.4 Measurement of antioxidant activity

#### 2.4.1 DPPH method

This study uses the method of Chuljerm et al., and the method description partly reproduces their wording (Chuljerm et al., 2023). In assay, DFP–RVT, DFP, RVT, and Trolox were dissolved in 50% (v/v) DMSO (0.2 mL) to achieve working concentrations (0–200 µM each) and then incubated with 0.4 mM DPPH radical (DPPH^•^) solution (0.2 mL) for 30 min, and A-values were photometrically measured at 517 nm against a blank reagent using a double-beam UV-Vis spectrophotometer (Shimadzu UV-1700 Series, Shimadzu Scientific Instruments, Nakagyo, Kyoto, Japan). In calculation: DPPH^•^-scavenging activity (%) was determined using the formula 1 - A_sample_/A_DMSO_ x 100. Accordingly, the activity is expressed as a half-maximal inhibitory concentration (IC_50_) value, in which a lower IC_50_ value indicates higher antioxidant activity.

#### 2.4.2 ABTS method

This study uses the method of Chuljerm et al., and the method description partly reproduces their wording (Chuljerm et al., 2023). Initially, the cationic ABTS radical (ABTS^•+^) solution was produced by oxidizing 7 mM ABTS with 2.45 mM potassium persulfate for 12–16 h and diluting the resulting ABTS^•+^ solution with deionized (DI) water to reach A-values of 0.700 ± 0.020 at 734 nm. In the assay, the DFP, DFP–RVT, and Trolox solution (0–200 µM each) were mixed with the ABTS^•+^ solution and incubated for precisely 6 min and photometrically measured at 734 nm against a blank reagent using a double-beam spectrophotometer. In calculation, ABTS^•+^ scavenging activity was determined using the formula 1–A_sample_/A_DMSO_ x 100. Accordingly, the activity is expressed as the IC_50_ value.

### 2.5 Assay of anti-hemolytic activity

This study uses the method of Chuljerm et al., and the method description partly reproduces their wording (Chuljerm et al., 2023). In principle, AAPH is an azo compound that can initiate oxidation reactions through nucleophilic and free radical mechanisms, consequently resulting in membrane lipid peroxidation and RBC lysis. In the assay, 20% red blood cell (RBC) suspension was previously prepared in PBS, pre-treated with DFP, RVT, DFP–RVT, and AA (0–200 µM each) or 1.5% DMSO (blank), and challenged with the 200 mM AAPH^•^ solution at 37 °C for 3 h. Afterward, the reaction mixture was diluted with PBS and centrifuged at 3000 g, and the supernatant was photometrically measured for A-values at 540 nm against a blank reagent using a double-beam spectrophotometer. Accordingly, inhibition of hemolysis (%) was determined using the formula 1–A_sample_/A_DMSO_ x 100.

### 2.6 Study of cytotoxicity

#### 2.6.1 Human hepatocyte culture

This study uses the method of Chuljerm et al., and the method description partly reproduces their wording (Chuljerm et al., 2023). Human hepatocellular carcinoma (Huh7) cells were cultured in DMEM supplemented with 10% (v/v) heat-inactivated FBS, 0.02% penicillin G, and 0.5% gentamicin at 37 °C under 5% atmospheric CO_2_ and adequate humidity and were harvested when reaching 80%–90% confluence.

#### 2.6.2 Preparation of human peripheral blood mononuclear cells

This study uses the method of Hutachok et al., and the method description partly reproduces their wording (Hutachok et al., 2021). Venous blood (10 mL) was drawn from blood donors, collected in sodium heparin (158 USP units)-coated tubes, and diluted with sterile PBS at a 1:1 (v/v) ratio. A sterile 50-mL conical tube containing Ficoll-Paque (Histopaque^®^-1077, a specific density of 1.077 g/mL) solution (15 mL), as per the manufacturer’s instruction, was gently overlaid on the diluted blood and centrifuged at 1,000 g, 4°C for 20 min. The plasma upper layer was aspirated. The peripheral blood mononuclear cells (PBMCs) at the interphase were carefully collected, transferred to a 15-mL sterile centrifuge tube, washed twice with PBS, gently sedimented by low-speed centrifugation at 4°C for 5 min, and resuspended in a complete culture medium for further study.

#### 2.6.3 MTT assay for cell viability

This study uses the MTT method of Hutachok et al., and the method description partly reproduces their wording (Hutachok et al., 2021). In other words, the assay is based on the ability of the mitochondrial reductase system in live cells to change the yellow-colored MTT dye to a blue-colored formazan product. In the assay, PBMCs (7 × 10^4^/well) and Huh7 cells (1 × 10^4^/well) were seeded into 96-well culture plates, treated with DFP–RVT (0–200 μM), and incubated in a humidified 5% CO_2_, 37 C incubator for 24 and 48 h. Then, the treated cells were washed twice with PBS and incubated with 5 mg/mL MTT solution for 4 h. Finally, the resulting blue formazan product was solubilized using 0.1 mL of DMSO, and the A-value was measured at a wavelength of 570 and 630 nm using a spectrophotometer. The viability of the treated cells was assessed by comparing the measured A-values without the treatment, which were considered 100% cell viability and calculated using the formula A_DFP_–_RVT_/A_DMSO_ x100.

### 2.7 Iron loading and treatment of HuH7 cells

Huh7 cells (2 × 10^5^/well) were cultured in the supplemented DMEM for 24 h for seeding (80% confluence) on culture plates, then switched to the DMEM supplemented with 1 mM FAC-rich solution, and further incubated for 8–10 h (Koonyosying et al., 2019). After washing twice with PBS, the iron-loaded cells were treated with DFP, DFP–RVT, RVT, and AA (0–50 M each) and incubated in a humidified 5% CO_2_, 37 C incubator for 24 and 48 h. The treated cells were then detached from the culture plate by trypsin/EDTA digestion and washed twice with PBS. The cellular iron, ROS, thiobarbituric acid-reactive substances (TBARS), GSH, GPX, and SOD activities were measured using the methods described below.

### 2.8 Assessment of iron-mobilizing activity

#### 2.8.1 Total non-heme iron

This study uses the ferrozine colorimetric method of Koonyosying et al. for assessing intracellular iron content, and the method description partly reproduces their wording (Koonyosying et al., 2019). Iron-loaded cells with the treatments were lysed with 200 mM NaOH overnight and neutralized with 10 mM HCl. Then, the cell lysate was mixed and incubated with an iron-releasing agent comprising 1.4 M HCl and 4.5% (w/v) KMnO_4_ in a 1:1 (v/v) ratio at room temperature for 2 h. Finally, the mixture was incubated with an iron chromogenic reagent containing 6.5 mM ferrozine, 6.5 mM neocuproine, 2.5 M ammonium acetate, and 1 M AA at room temperature for 30 min, and the A-value was photometrically measured at 562 nm against a blank reagent. The liver iron content was normalized with protein content in the lysate, as described below and expressed as μg iron/mg of protein.

#### 2.8.2 Redox-active iron

This study uses a fluorescence technique, and the method description partly reproduces their wording (Mei et al., 2020; Tam et al., 2022). FerroOrange is a sensitive iron-binding fluorescent reporter that has been recently used to assess redox-active iron content in cells. An amount of 24 μg of FO powder was reconstituted with 35 μL of DMSO to achieve a stock FO solution (1 mM), which was subsequently further diluted with DMEM to prepare a working FO solution (1 μM). In the assay, iron-loaded cells with the treatments were incubated with 1 μM FO for 30 min, and fluorescence intensity (FI) was measured using a confocal multiplex microscope (Zeiss LSM 900, Carl Zeiss Microscopy GmbH, Jena, Germany) (λ_excitation_ 561 nm/λ_emission_ 570 nm).

#### 2.8.3 Protein content

The cell lysate was dissolved in PBS (1:1, v/v) and mixed with Bradford reagent (100:1, v/v). The mixture was then incubated at room temperature for 30 min. Finally, the A-value of the colored product was measured at 595 nm against the reagent blank by spectrophotometry. A standard BSA solution (0–500 μg/mL) was used to make a standard graph for the determination of the membrane protein concentration (Settakorn et al., 2022).

### 2.9 Determination of ROS-scavenging activity

This study uses the method of Koonyosying et al., and the method description partly reproduces their wording (Koonyosying et al., 2019). In principle, cytosolic esterases hydrolyze 2′,7′-dichlorofluorescein diacetate (DCFH-DA) to generate 2′,7′-dichlorofluorescein (DCFH), which will be subsequently oxidized by ROS to form a DCF product yielding vivid green FI. Herein, iron-loaded cells were incubated with DCFH-DA (2 μM) for 30 min and washed twice with PBS, and FI was measured using a 96-well plate spectrofluorometer (λ_excitation_ 485 nm/λ_emission_ 535 nm).

### 2.10 Determination of anti-lipid peroxidation activity

This study uses the method of Koonyosying et al., and the method description partly reproduces their wording (Koonyosying et al., 2018). Lipid peroxidation products, including malondialdehyde (MDA), quantitatively react with TBA and form pink-colored TBARS. In assay, iron-loaded cells with the treatments were lysed by ultrasonication, and the cell lysate was incubated with a mixture of 1% MPA and 0.67% (w/v) TBA at 95°C for 60 min. After cooling, n-butanol was added to the mixture, and the butanol phase was subjected to measure the A-value at 540 nm using a spectrophotometer.

### 2.11 Detection of antioxidant defense properties

#### 2.11.1 GSH content

This study measures GSH based on the method established by Jagetia et al., and the method description partly reproduces their wording (Jagetia et al., 2004). The measurement of GSH concentration was determined, according to the manufacturer’s instructions. In brief, the cell lysate was deproteinized by 25% (w/v) trichloroacetic acid and centrifuged. The supernatant was collected, mixed with 0.2 M PBS pH 8.0 solution, and reacted with Ellman’s reagent (0.06 mM DTNB) at room temperature for 10 min, and the A-value was photometrically measured at 412 nm against a blank reagent.

#### 2.11.2 GPX activity

This study assays GPX activity based on the method established by Jagetia et al., and the method description partly reproduces their wording (Jagetia et al., 2004). An assay of GPX activity was performed according to the manufacturer’s instructions. In brief, the cell lysate was mixed and incubated with a reagent containing glutathione reductase, GSH, and NADPH_2_ at 37 °C for 5 min. The reaction mixture was then challenged with cumene hydroperoxide, and the A-value was recorded at 340 nm against a blank reagent using a double-beam UV-Vis spectrophotometer.

#### 2.11.3 SOD activity

This study uses the method of Jagetia et al., and the method description partly reproduces their wording (Jagetia et al., 2004). Herein, SOD activity was assayed according to the manufacturer’s instruction using xanthine oxidase for catalyzing oxidation of xanthine to uric acid and superoxide radical (O_2_^•-^) and a slightly red water-soluble tetrazolium 1 (4-[3-(4-Iodophenyl)-2-(4-nitrophenyl)-2H-5-tetrazolio]-1,3-benzene sulfonate) or WST-1 substrate, accepting an electron from the resulting O_2_^•-^ and forming a dark red formazan product. Accordingly, the IC_50_ values of the SOD or SOD-like substance activities were determined.

### 2.12 Statistical analysis

Results and statistical significance were analyzed using SPSS 22.0 Program for Windows, and the data are presented in mean ± standard deviation (SD). Group comparisons were conducted through an analysis of variance (ANOVA) test, followed by an LSD *post hoc* test. A *p*-value <0.05 is considered a significant difference.

## 3 Results

### 3.1 Iron-binding activity

**FIGURE 1:**
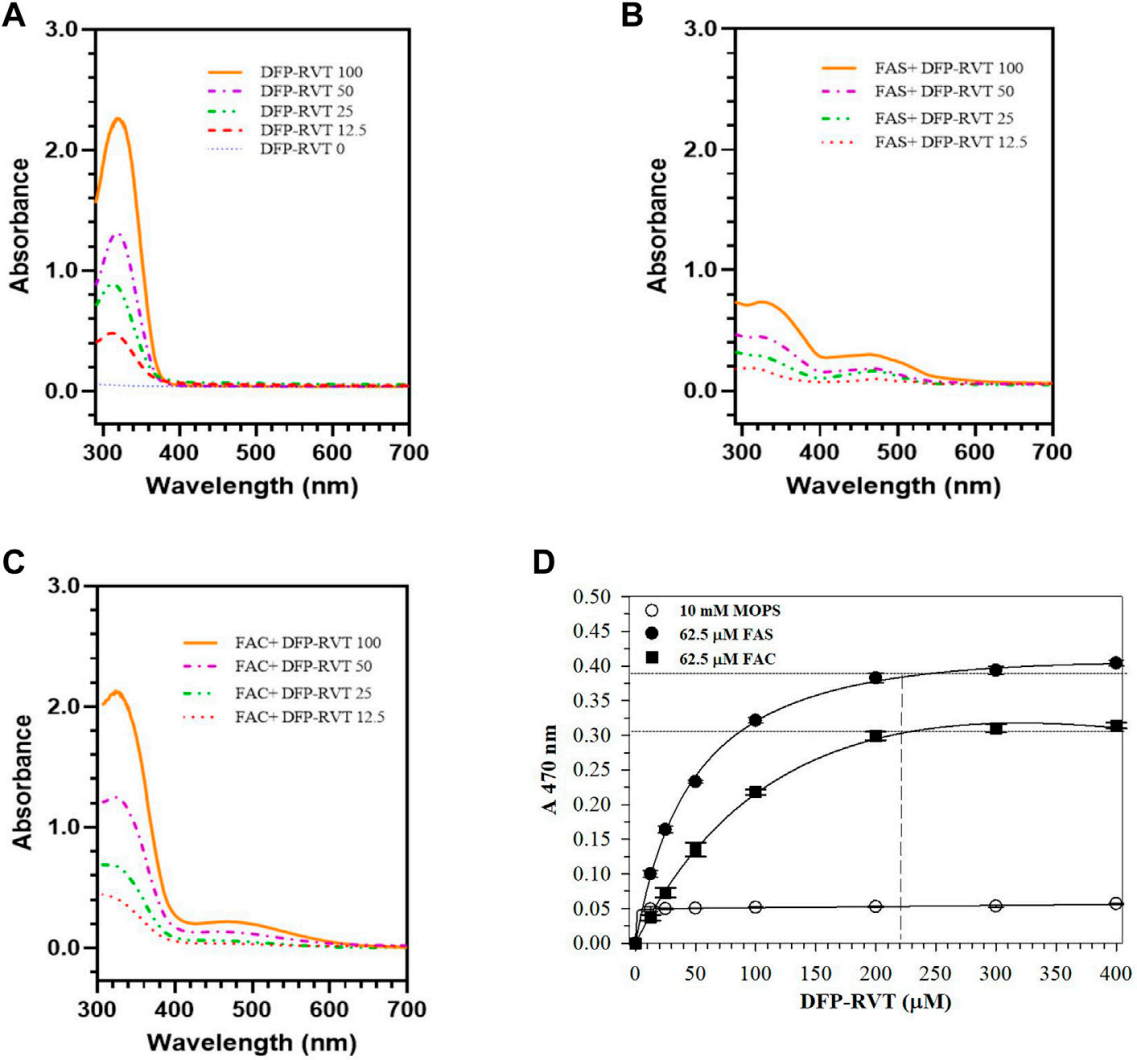
Spectral profile of DFP-RVT alone **(A)**, Fe(II)-DFP-RVT **(B)** and Fe(III)-DFP-RVT **(C)** products, and saturation curves of Fe(II)- and Fe(III)-DFP-RVT products **(D)**. DFP–RVT solution (12.5–100 μM) was incubated with FAS or FAC solution (62.5 μM), and the A-values were photometrically measured at the wavelengths of 200–800 nm against a blank reagent. Abbreviations: A, absorbance; DFP, deferiprone; DFP–RVT, deferiprone–resveratrol hybrid; FAC, ferric ammonium citrate; FAS, ferrous ammonium sulfate; RVT, resveratrol.

In the finding, DFP–RVT *per se* showed light absorption at the UV region (λ_max_ = 340 nm) exerted by the pyridine ring moiety (Figure 1A). Apparently, the compound bound Fe^2+^ (FAS solution) and Fe^3+^ (FAC solution) rapidly in a concentration-dependent manner, forming a red-color iron-(DFP–RVT) complex and giving a dominant absorption peak (λ_max_ = 470 nm) (Figures 1B, C, respectively). Accordingly, the iron (62.5 μmol/L) increasingly bound to DFP–RVT (12.5 → 400 μmol/L) and reached saturation points at 210 μmol/L DFP–RVT (Figure 1D), indicating an estimated 3:1 molar ratio of DFP–RVT to iron.

### 3.2 Antioxidant activity

**FIGURE 2:**
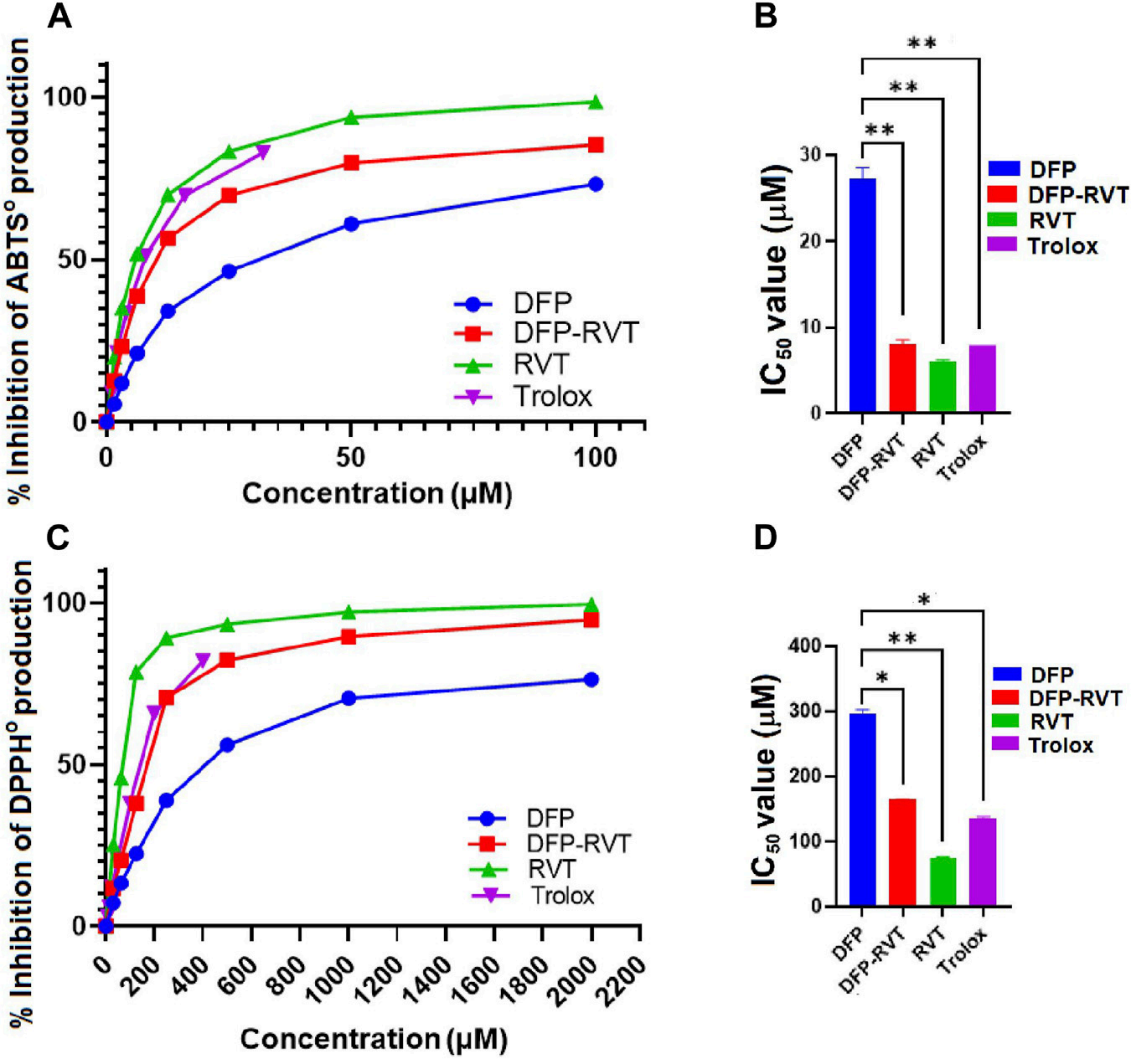
Antioxidant activity and IC_50_ values of DFP, DFP–RVT, RVT, and Trolox. The compounds were assayed for inhibitory effects on radical production by using ABTS **(A, B)** and DPPH **(C, D)** methods, and their IC_50_ values were determined from the inhibition curves. Data obtained from three independent triplicate experiments are expressed as mean ± SD. Accordingly, ^*^ *p* < 0.05 and ^**^ *p* < 0.01 when compared with DFP. Abbreviation: ABTS, 2,2′-azino-bis (3-ethylbenzthiazoline-6-sulfonic acid) diammonium; DFP, deferiprone; DFP–RVT, deferiprone–resveratrol hybrid; DPPH, 2,2-diphenyl-1-picrylhydrazyl; IC_50_, half-maximal inhibitory concentration; RVT, resveratrol; Trolox, 6-hydroxy-2,5,7,8-tetramethylchroman-2-carboxylic acid.

By using the ABTS method, DFP, DFP–RVT, RVT, and Trolox inhibited the production of ABTS^•^ in concentration-dependent manners with different efficiencies (Figure 2A). Considering their IC_50_ values (Figure 2B), DFP–RVT (IC_50_ = 8.0 ± 0.6 μM) showed lower ABTS^•^-scavenging activity than DFP (IC_50_ = 27.2 ± 1.3 μM), higher than RVT (IC_50_ = 6.0 ± 0.3 μM) (*p* < 0.01), and as effective as Trolox (IC_50_ = 7.9 ± 0.1 μM), suggesting that the antioxidant potency would be RVT > DFP–RVT ∼ Trolox > DFP. Similarly, by using the DPPH method, all four compounds inhibited the production of DPPH^•^ in concentration-dependent manners with different efficiencies (Figure 2C). Considering their IC_50_ values, DFP–RVT (IC_50_ = 164 ± 1 μM) showed significantly higher DPPH^•^-scavenging activity than DFP (IC_50_ = 295.3 ± 6.8 μM), lower than RVT (IC_50_ = 75.8 ± 1.0 μM), and was equal to Trolox (IC_50_ = 135.1 ± 2.8 μM) (Figure 2D), suggesting that the antioxidant potency would be RVT > DFP–RVT ∼ Trolox > DFP.

### 3.3 Inhibitory effect of RBC hemolysis

**FIGURE 3:**
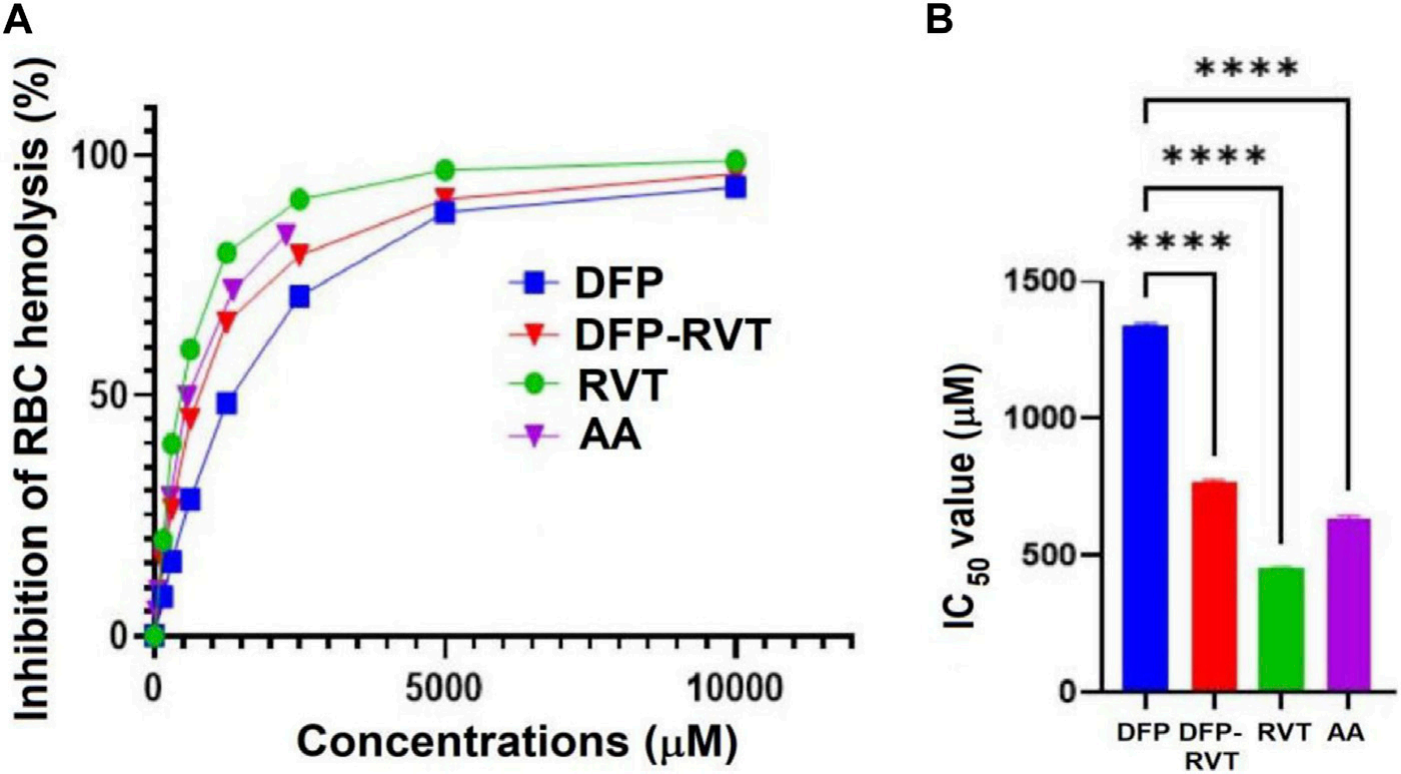
Percentage **(A)** and IC_50_ values **(B)** for the anti-hemolytic activity of DFP, DFP–RVT, RVT, and AA on RBCs induced by AAPH^•^. RBC suspension was initially challenged with the AAPH^•^ solution and treated with the tested compounds (12.5–1,000 µM each), and the A-values were photometrically measured at 540 nm. Data obtained from three independent triplicate experiments are expressed as mean ± SD. Accordingly, ^****^ *p* < 0.01 when compared with DFP. Abbreviations: A, absorbance; AA, L-ascorbic acid; DFP, deferiprone; DFP–RVT, deferiprone–resveratrol hybrid; IC_50_, half-maximal inhibitory concentration; RBC, red blood cell; RVT, resveratrol; Trolox, 6-hydroxy-2,5,7,8-tetramethylchroman-2-carboxylic acid.

In the finding, AA, DFP–RVT, DFP, and RVT significantly inhibited the hemolysis of RBC induced by AAPH^•^ in a concentration-dependent manner (Figure 3A), with IC_50_ values of 455 ± 2, 640 ± 7, 771 ± 7, and 1340 ± 9 μM, respectively (Figure 3B). Accordingly, degrees of inhibition were RVT > AA > DFP–RVT > DFP.

### 3.4 Cytotoxic effect

**FIGURE 4:**
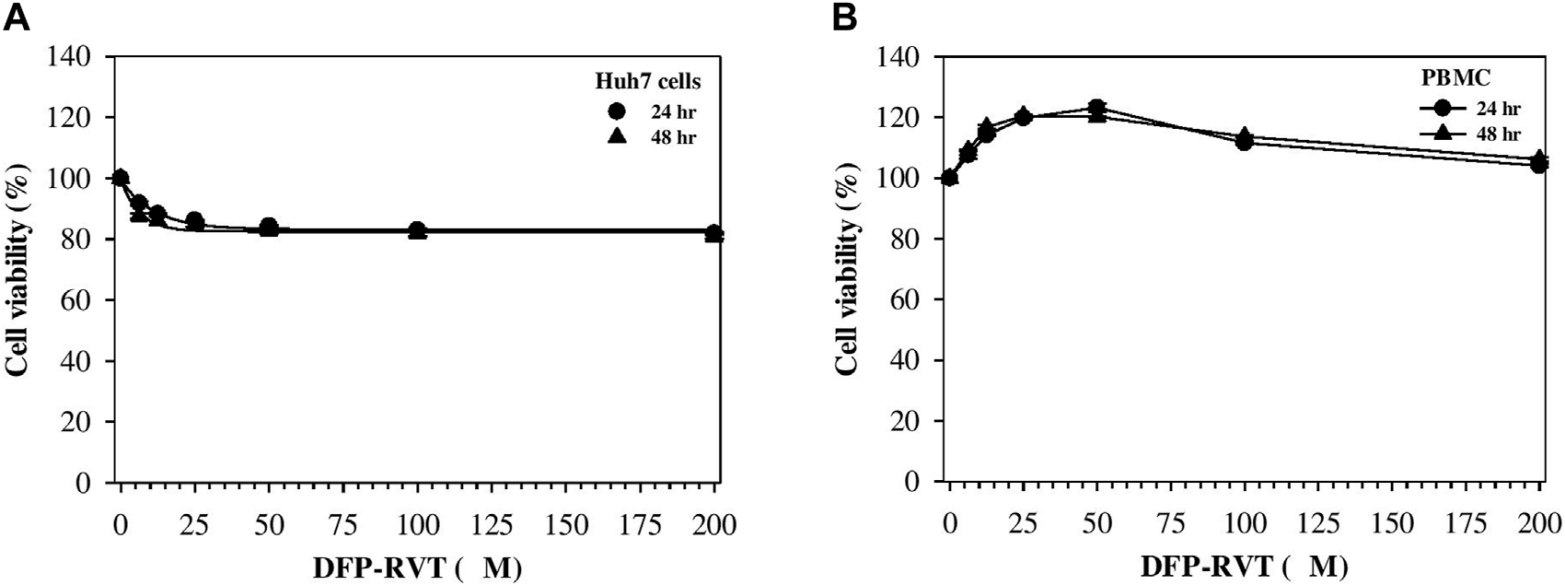
Viability of Huh7 cells **(A)** and PBMCs **(B)** treated with DFP–RVT. The cells were treated with DFP–RVT (0–200 μM) for 24 and 48 h, and their viability was determined by colorimetric MTT assay. Data obtained from three separate sets of triplicate experiments are expressed as mean ± SD. Abbreviations: DFP–RVT, deferiprone–resveratrol hybrid; Huh7, human hepatocellular carcinoma; MTT, 3-(4,5-dimethylthiazol-2-yl)-2,5-diphenyl tetrazolium bromide); PBMCs, peripheral blood mononuclear cells.

In the finding, DFP–RVT treatment for 24 and 48 h slightly decreased the viability of human hepatocellular carcinoma Huh7 cells in a concentration-dependent manner at 6.25–25 μM and did not further decrease the viability when increased up to 200 μM, but the viability was not <80% (Figure 4A). In contrast, DFP–RVT treatments (12.5–50 µM) slightly increased the viability of normal PBMCs in a concentration-dependent manner, and the viability was restored to the original level when DFP–RVT treatments at 50–200 µM were used (Figure 4B). The findings suggest that DFP–RVT would not be harmful to human hepatic Huh7 cells and normal human PBMCs.

### 3.5 Cellular iron-mobilizing property

**FIGURE 5:**
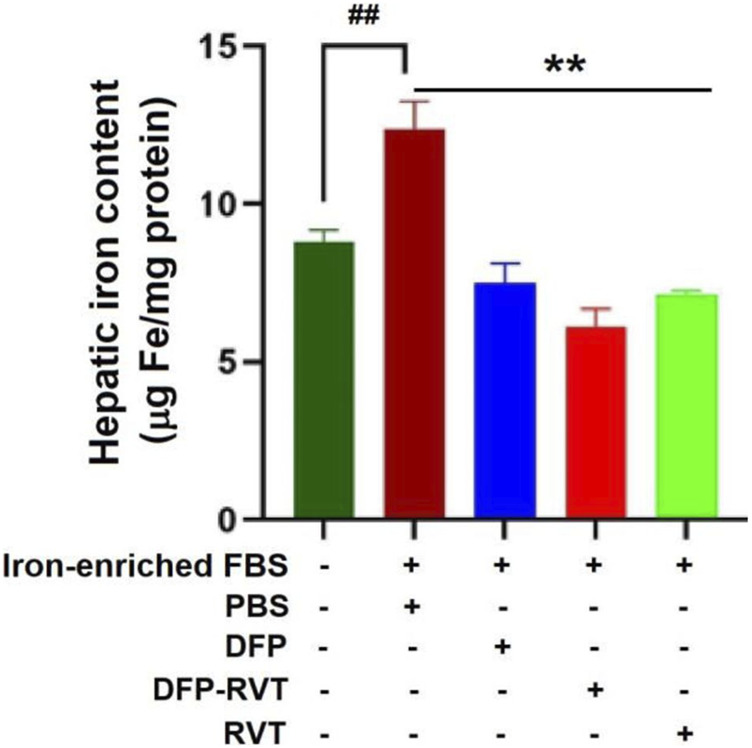
Iron-mobilizing activity of DFP–RVT in Huh7 cells assayed by ferrozine colorimetry. Huh7 cells were incubated in DMEM without or with iron-enriched FBS and treated with DFP, DFP–RVT, and RVT (50 µM each), and total iron was measured using a ferrozine chromogen. Data obtained from three independent triplicate experiments are expressed as mean ± SD. Accordingly, ^##^ *p* < 0.01 when compared without iron-enrich FBS; ^**^ *p* < 0.01 when compared with PBS. Abbreviations: DFP, deferiprone; DFP–RVT, deferiprone–resveratrol hybrid; DMEM, Dulbecco’s modified Eagle's medium; FBS, fetal bovine serum; PBS, phosphate-buffered saline; RVT, resveratrol.

**FIGURE 6:**
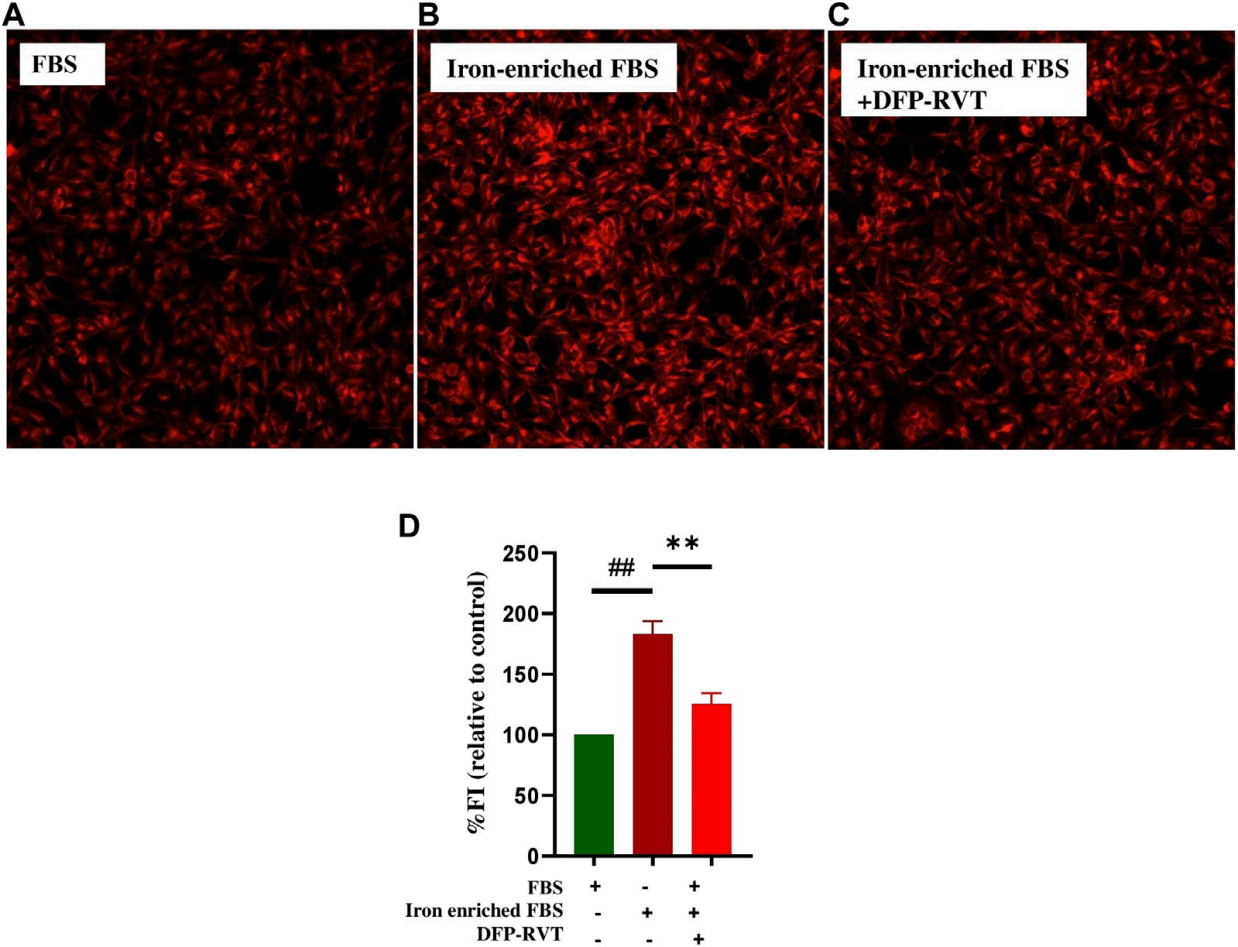
Iron-mobilizing activity of DFP-RVT in Huh7 cells. Huh7 cells were incubated in FBS **(A)**, iron-enriched FBS **(B)**, and iron-enriched FBS treated with DFP-RVT **(D)**, and labeled with FO fluorogenic probe. Redox-active iron was detected by fluorometry. Data obtained from three independent triplicate experiments are expressed as mean ± SD. Accordingly, ^##^ *p* < 0.01 when compared without iron-enriched FBS; ^**^ *p* < 0.01 when compared with PBS. Abbreviations: DFP–RVT, deferiprone–resveratrol hybrid; DMEM, Dulbecco’s modified Eagle's medium; FO, FerroOrange; FBS, fetal bovine serum; PBS, phosphate-buffered saline.

The results obtained from a ferrozine method and shown in Figure 5 demonstrated that the iron content was considerably increased in Huh7 cells incubated in DMEM with included iron-enriched FBS (*p* < 0.01) when compared without inclusion of the iron-enriched FBS. Importantly, the iron content in the iron-loaded cells was remarkedly decreased by treatments with DFP, DFP–RVT, and RVT (50 µM each) (*p* < 0.001) when compared with PBS treatment. Proposedly, the efficiency of the chelation would be in the order of DFP–RVT > RVT > DFP. Consistently, by detecting LIPs with FO fluorochrome, microscopic illustration has shown even more intense FI in Huh7 cells incubated in DMEM supplemented with iron-enriched FBS (Figure 6A) than those without the iron-FBS (Figure 6B), and FI was lowered by 50 µM DFP–RVT treatment (Figure 6C). As shown in a bar graph (Figure 6D), the level of LIP was stoichiometrically increased in Huh7 cells incubated in DMEM supplemented with iron-enriched FBS (*p* < 0.01) when compared with those without the iron-FBS, and it was decreased significantly by 50 µM DFP–RVT treatment when compared with PBS treatment (*p* < 0.01). The two results confirm that DFP–RVT effectively chelates non-heme iron and redox-active LIPs in iron-overloaded liver cells.

### 3.6 Cellular ROS-scavenging activity

**FIGURE 7:**
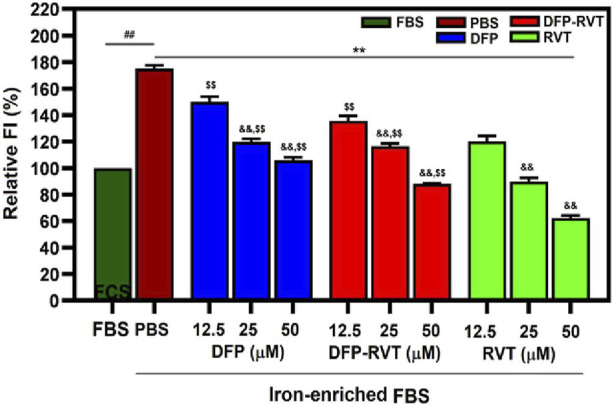
Cellular ROS-scavenging activities of DFP–RVT, DFP, RVT, and AA treatments in iron-loaded Huh7 cells. Huh7 cells were incubated in DMEM without or with iron-enriched FBS and treated with DFP, DFP–RVT, and RVT (12.5–50 µM each). ROS was detected with DCF, and FI was measured by fluorometry. Data obtained from three independent triplicate experiments are expressed as mean ± SD. Accordingly, ^##^ *p* < 0.01 when compared with FBS; ^**^ *p* < 0.01 when compared with PBS; ^&&^ *p* < 0.01 when compared with 12.5 μM concentration; ^$$^ *p* < 0.01 when compared with RVT at equal concentrations. Abbreviations: DCF, dichlorofluorescein; DFP, deferiprone; DFP–RVT, deferiprone–resveratrol hybrid; FBS, fetal bovine serum; FI, fluorescence intensity; DMEM, Dulbecco’s modified Eagle's medium; PBS, phosphate-buffered saline; ROS, reactive oxygen species; RVT, resveratrol.

Remarkably, the ROS level was increased in Huh7 cells incubated with iron-enriched FBS (*p* < 0.01) when compared with FBS alone (Figure 7). Afterward, the increased ROS level was diminished by DFP–RVT, DFP, and RVT treatments in concentration-dependent manners (*p* < 0.01) when compared with PBS treatment, in which the degree of the ROS-scavenging effect was RVT > DFP–RVT > DFP at equivalent concentrations.

### 3.7 Anti-lipid peroxidation activity

**FIGURE 8:**
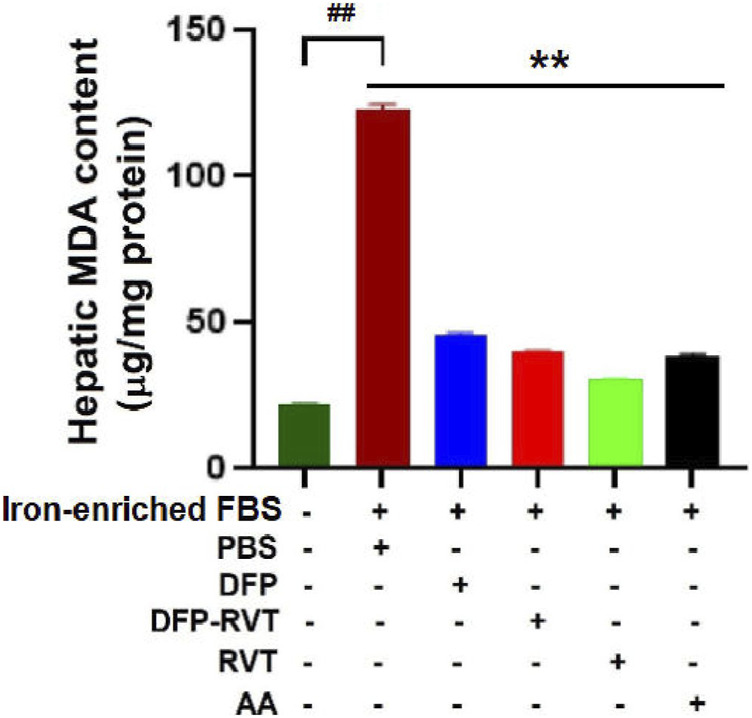
Anti-lipid peroxidation activities of DFP–RVT, DFP, RVT, and AA in iron-loaded Huh7 cells. Huh7 cells were incubated in DMEM without or with iron-enriched FBS and treated with DFP, DFP–RVT, RVT, and AA (50 µM each), and the amount of lipid peroxidation products as MDA was measured using a colorimetric TBARS assay. Data obtained from three independent triplicate experiments are expressed as mean ± SD. Accordingly, ^##^ *p* < 0.01 when compared without iron-enriched FBS, ^**^ *p* < 0.01 when compared with PBS. Abbreviations: AA, ascorbic acid; DFP, deferiprone; DFP–RVT, deferiprone–resveratrol; DMEM, Dulbecco’s modified Eagle's medium; FAC, ferric ammonium citrate; FBS, fetal bovine serum; MDA, malondialdehyde; PBS, phosphate-buffered saline; RVT, resveratrol; TBARS, thiobarbituric acid-reactive substances.

In iron-loaded cells, ROS generated by iron catalysis can oxidize lipid molecules, particularly polyunsaturated fatty acid (PUFA), to form lipid peroxide and lipid hydroperoxide (LPO). Herein, the level of LPO products such as MDA was found to significantly increase in Huh7 cells incubated with iron-enriched FBS when compared with those without the iron-FBS treatment (Figure 8). Interestingly, the increased MDA level was dramatically reduced by DFP–RVT, DFP, RVT, and AA treatments when compared with PBS treatment, suggesting their anti-lipid peroxidation activities, in which the RVT seemed to be the most effective among these compounds.

### 3.8 Cellular antioxidant responsiveness

**FIGURE 9:**
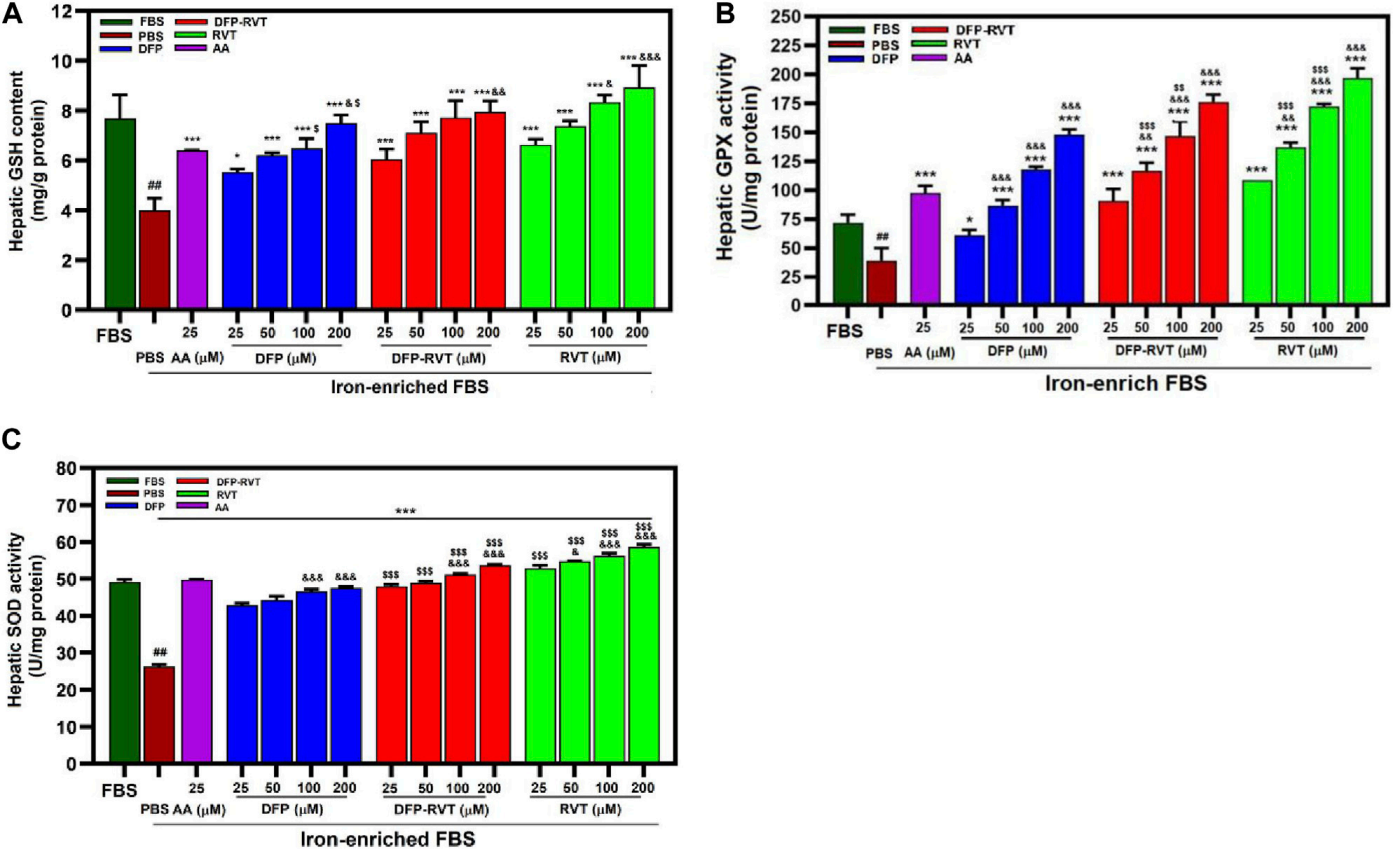
Levels of cellular GSH content **(A)**, GPX **(B)**, and SOD **(C)** activities in iron-loaded Huh7 cells treated with DFP–RVT, DFP, RVT, and AA. Cells were incubated in DMEM without or with iron-enriched FBS and treated with DFP, DFP–RVT and RVT (25–200 µM each), and AA (25 µM each). The GSH concentration and GPX and SOD activities were determined. Data obtained from three independent triplicate experiments are expressed as mean ± SD. Accordingly, ^##^ *p* < 0.01 when compared without iron-enriched FBS; ^*^ *p* < 0.05 and ^**^ *p* < 0.01 when compared with PBS; ^&&&^ *p* < 0.01 when compared with 25 μM concentration; ^$$$^ *p* < 0.01 when compared at the equal concentrations. Abbreviations: AA, ascorbic acid; DFP, deferiprone; DFP–RVT, deferiprone–resveratrol hybrid; FBS, fetal bovine serum; GSH, reduced glutathione; GPX, glutathione peroxidase; DMEM, Dulbecco’s modified Eagle's medium; PBS, phosphate-buffered saline; RVT, resveratrol; SOD, superoxide dismutase.

Our findings have revealed that levels of GSH content and GPX and SOD activities were decreased in Huh7 cells incubated in iron-enriched FCS consistently and significantly when compared with FBS (Figures 9A–C, respectively). Interestingly, the levels of the decreases were restored and increased significantly by DFP–RVT, DFP, and RVT in concentration-dependent manners and by 25 μM AA when compared with PBS, in which RVT was slightly more effective than the other compounds at equivalent concentrations.

## 4 Discussion

Iron chelators find extensive use in the medical treatment of individuals afflicted with iron overload (Adjimani and Asare, 2015). In addition, some iron chelators may contribute to a decrease in the generation of ROS, which otherwise inflicts damage upon crucial cellular components (Jomova and Valko, 2011; Adjimani and Asare, 2015; Mobarra et al., 2016). For instance, DFP itself is a bidentate iron chelator used to remove excessive iron depositions in individuals with iron overload (particularly thalassemia), and its antioxidant property is able to scavenge radicals in the oxidative iron-induced hepatocytes, which was more potent than DFO (Morel et al., 1992; Crosland, 1995; Moridani and O'Brien, 2001). In supplementation, the administration of exogenous synthetic and natural antioxidants is required to increase and improve the generation and activation of endogenous antioxidant mechanisms (Kontoghiorghes and Kontoghiorghe, 2019). In a medical regimen, combining two different iron chelators and adjunctive treatment of one chelator with one antioxidant or antioxidative iron chelator could promote more efficiency in preventing the redox cycling of Fe^2+^, antioxidant activity, and ROS-scavenging properties than their monotherapy. Accordingly, strategic therapy can lead to the development of drug candidates or prodrugs with co-adjuvant roles in oxidative stress-dependent pathologies (Chaves et al., 2012). For instance, the DFP prodrug, antioxidants such as butyrate hydroxytoluene (BHT), resveratrol, and covalent linkers such as poly(ethylene)glycol or amines are utilized for synthesis and water solubilization of dual antioxidant/iron chelator hybrids like DFP-BHT (Bebbington et al., 2002) and DFP–RVT (Xu et al., 2017).

In this study, DFP–RVT can bind Fe(II) and Fe(III) rapidly in a concentration-dependent manner, forming a red Fe-(DFP–RVT)_3_ complex with λ_max_ at 470 nm, where a molar ratio of the DFP–RVT to both of the iron would be 3:1. In comparison, a red Fe-(DFP)_3_ complex showed an absorption peak with λ_max_ at approximately 500 nm (Sooriyaarachchi and Gailer, 2010; Sebestik et al., 2012; Timoshnikov et al., 2020). RVT (3,5,4′-trans-trihydroxystilbene) is a polyphenolic compound widely distributed in food and edible plants. Interestingly, it can bind and reduce Fe^3+^ to Fe^2+^ and exhibits more efficient anti-lipid peroxidation activity against Fe^2+^-induced phosphatidylcholine liposomes than Trolox but weaker activity against APPH^•^-induced liposomes (Tadolini et al., 2000). In contrast, its analog, 3,4,4 -trihydroxy-trans-stilbene, was far more efficient than RVT in protecting against ROS-induced lipid peroxidation and hemolysis of human RBCs induced by AAPH^•^ (Cai et al., 2011). By modification of the RVT structure, synthetic RVT analogs such as polyhydroxystilbenes were found to be more potent in anti-lipid peroxidation in liver homogenates and anti-RBC hemolysis than RVT in rats (Lu et al., 2002). Moreover, Mikstacka and others reported the IC_50_ values of anti-hemolysis of AAPH^•^-induced human RBCs by trans-RVT, quercetin, and pterostilbene to be 11.3 +/− 2.9, 16.1 +/− 0.9, and 10.8 +/− 5.9 µM, respectively (Mikstacka et al., 2010). Our recent evidence has elucidated that DFP–RVT had stronger anti-RBC hemolysis, anti-membrane lipid peroxidation, and hepatoprotective properties in *P. berghei*-infected mice than DFP (Chuljerm et al., 2021). Consistently, the present study showed an inhibitory effect of DFP–RVT against hemolysis of RBCs induced by AAPH^•^ due to the anti-lipid peroxidation on the RBC membrane. Furthermore, DFP–RVT could permeate cell membranes more readily and have more bioavailability in the body than DFP due to its higher partition coefficient value (octanol/water) than DFP (Xu et al., 2017; Maneekesorn et al., 2021).

The affinity constant, pFe(III) value, which results from the extensive delocalization of electrons in resonance structures, of potential chelators for binding iron(III) can be determined by the automatic spectrophotometric titration. In this case, Xu et al. reported the pFe(III) values of DFP–RVT and DFP were 20.6 and 19.6, respectively, indicating stronger metal-chelating activity than DFP (Xu et al., 2017). In Jiang et al.’s work, DFP hybrids were synthesized by conjugating 3-hydroxypyridine-4-one amide derivatives with coumarin derivatives, of which the three products [pFe(III) values = 18.93, 18.98, and 20.16, respectively] possessed iron-chelating activities superior to DFP [pFe(III) = 17.50], ROS-scavenging, and monoamine oxidase B inhibitory effects (IC_50_ = 87.9 and 98.6 nM) superior to the reference drug pargyline (IC_50_ = 107.3 nM) (Jiang et al., 2020). Our investigation has demonstrated that DFP–RVT was neither toxic to PBMC nor human Huh7 hepatic cell lines even used at concentrations up to 200 µM.

Even with or without iron overload, oxidative stress in liver injury is a major pathogenetic factor in the progress of liver fibrosis. Biochemically, ROS emerge from cellular processes like mitochondrial electron transport, neutrophil actions, and non-enzymatic iron catalysis involving the Haber–Weiss and Fenton reactions (Ramezanpour et al., 2019). In turn, excessive ROS can threaten functional biomolecules, such as PUFA, nucleic acids, and proteins, in living cells and organisms (Koonyosying et al., 2019). Pathologically, oxidative stress within the liver can induce the progression of liver diseases, including non-alcoholic fatty liver disease (NAFLD), liver inflammation, and hepatocellular carcinoma associated with hepatitis C infection (Kurhaluk et al., 2017). Surprisingly, consumption of RVT inhibited lipid peroxidation, increased the levels of GPX and SOD activities, and alleviated the infiltration of inflammatory cells and fibrosis of liver tissue of dimethylnitrosamine-induced rats (Lee et al., 2010). In action, RVT proficiently donates electron(s) from its hydroxyl groups within the molecule to quench DPPH^•^ (He et al., 2018). In other words, TBARS, including MDA, serve as quantifiable end-products, resulting from lipid peroxidation reactions, and are frequently used as biomarkers for oxidative stress (Breusing et al., 2010). In findings, we have revealed that DFP–RVT restored the increased levels of iron deposition, labile redox-active iron, ROS, and MDA in iron-loaded Huh7 hepatic cells, which was more potent than DFP, suggesting the iron-chelating, ROS-scavenging, and anti-lipid peroxidation activities.

Regarding antioxidant defense in the body, levels of biomarkers such as GSH, SOD, GPX, and CAT activities are proposedly measured (He et al., 2018). Their plasma and cellular contents contribute to the total antioxidant capacity (Schumann et al., 2005; Li et al., 2019). Cellular GSH *per se* donates its electron (H^−^) to couple with a singlet H^−^ in free radicals and prevent the attraction of H^−^ from the biomolecules. In addition, cellular SOD functions to facilitate the coupling of O_2_^−•^ and 2H^+^ to form H_2_O_2_, which will be subsequently destroyed to water by GPX and CAT catalysis (Kurhaluk et al., 2017). Wang and others found that RVT treatment improved alanine aminotransferase and aspartate aminotransferase activities and decreased the MDA content, GSH content, and GSH activity levels in the livers of mice induced by iron dextran (10 mg/kg, intraperitoneally) (Wang et al., 2022). Oral administration of DFP can increase the level of GSH but neither GPX nor CAT in the liver tissue of TMH–ferrocene-fed mice (Eybl et al., 2002). In addition, DFP treatment was found to attenuate and increase hepatic GPX activity in iron-loaded liver damage in tamoxifen-induced rats (Cerna et al., 2011). In the rat liver incubated with DFP, GSH, and N-acetylcysteine (NAC), not only the DFP–diglucuronide conjugate but also DFP–GSH and DFP–NAC metabolites were produced by the catalysis of microsomal cytochrome P450 enzymes (Zheng et al., 2021). More importantly, treatments of DFP, naringin, quercetin, and myricetin can increase the GSH level and stimulate the activity of selenoenzymes, GPX, and thioredoxin in the liver of Wistar rats (Zheng et al., 2021). In limitation, Huh7 and HepG2 hepatic cell lines are claimed as human hepatocellular carcinoma cells, but they have some characteristics and biological behavior different from normal liver cells. We lack high-throughput HPLC coupled with mass spectrometry (MS) or quadrupole time-of-flight/MS, so we did not measure metabolites present in the Huh7 hepatic cells incubated with DFP–RVT.

Taken together, DFP–RVT is a mitigation of oxidative stress and inflammation in liver cells under iron overload, providing ROS-scavenging, iron-chelating, anti-membrane lipid peroxidation, and antioxidant defense-modulating properties. Although this study adds to our understanding of DFP–RVT’s properties, it could benefit from further differentiation and exploration *in vivo* to fully showcase its potential impact on liver health. The bioactivity superiority of DFP–RVT to DFP will drive further experiments to be investigated in beta-knockout β-thalassemic mice with iron overload induced by intraperitoneal injection of iron dextran and feeding ferrocene-supplemented diet.

## 5 Conclusion

Our preliminary assays have highlighted a distinct advantage of DFP–RVT as a potential iron chelator because it exerts antioxidant and iron-chelating activities. It not only effectively extracts iron from iron-loaded cells while maintaining the viability of human hepatocytes. It also mobilizes cellular iron, diminishes ROS levels, protects against oxidative damage, and stimulates the antioxidant defense system in hepatocytes with iron overload. This implies its safe suitability for therapeutic use and promises characteristic positions DFP–RVT as a valuable contender in creating novel iron-chelating agents and various other medical applications.

## Data Availability

The raw data supporting the conclusion of this article will be made available by the authors, without undue reservation.
